# VCE-003.2, a novel cannabigerol derivative, enhances neuronal progenitor cell survival and alleviates symptomatology in murine models of Huntington’s disease

**DOI:** 10.1038/srep29789

**Published:** 2016-07-19

**Authors:** Javier Díaz-Alonso, Juan Paraíso-Luna, Carmen Navarrete, Carmen del Río, Irene Cantarero, Belén Palomares, José Aguareles, Javier Fernández-Ruiz, María Luz Bellido, Federica Pollastro, Giovanni Appendino, Marco A. Calzado, Ismael Galve-Roperh, Eduardo Muñoz

**Affiliations:** 1 Instituto Ramón y Cajal de Investigación Sanitaria (IRYCIS), Ctra, Colmenar Viejo, km. 9100, Madrid, Spain; 2 Departamento de Bioquímica y Biología Molecular I, Universidad Complutense, Madrid, Spain; 3 Centro de Investigación Biomédica en Red sobre Enfermedades Neurodegenerativas (CIBERNED), Madrid, Spain; 4 VivaCell Biotechnology España, Córdoba, Spain; 5 Instituto Maimónides de Investigación Biomédica de Córdoba, Hospital Universitario Reina Sofía, Departamento de Biología Celular, Fisiología e Inmunología, Universidad de Córdoba, Spain; 6 Instituto Universitario de Investigación en Neuroquímica, Departamento de Bioquímica y Biología Molecular III, Universidad Complutense, Madrid, Spain; 7 Dipartimento di Scienze del Farmaco, Università del Piemonte Orientale, Novara, Italy

## Abstract

Cannabinoids have shown to exert neuroprotective actions in animal models by acting at different targets including canonical cannabinoid receptors and PPARγ. We previously showed that VCE-003, a cannabigerol (CBG) quinone derivative, is a novel neuroprotective and anti-inflammatory cannabinoid acting through PPARγ. We have now generated a non-thiophilic VCE-003 derivative named VCE-003.2 that preserves the ability to activate PPARγ and analyzed its neuroprotective activity. This compound exerted a prosurvival action in progenitor cells during neuronal differentiation, which was prevented by a PPARγ antagonist, without affecting neural progenitor cell proliferation. In addition, VCE-003.2 attenuated quinolinic acid (QA)-induced cell death and caspase-3 activation and also reduced mutant huntingtin aggregates in striatal cells. The neuroprotective profile of VCE-003.2 was analyzed using *in vivo* models of striatal neurodegeneration induced by QA and 3-nitropropionic acid (3NP) administration. VCE-003.2 prevented medium spiny DARPP32^+^ neuronal loss in these Huntington’s-like disease mice models improving motor deficits, reactive astrogliosis and microglial activation. In the 3NP model VCE-003.2 inhibited the upregulation of proinflammatory markers and improved antioxidant defenses in the brain. These data lead us to consider VCE-003.2 to have high potential for the treatment of Huntington’s disease (HD) and other neurodegenerative diseases with neuroinflammatory traits.

Cannabinoids the main active compounds of marijuana (*Cannabis sativa*), and its endogenous counterparts anandamide and 2-arachidonoyl glycerol have attracted the interest of the scientific community in the last decade owing to their prominent effects on neurodegenerative and neuroinflammatory conditions^1^. Cannabinoids exert neuroprotective actions in various experimental models of neurodegenerative diseases, and most of their effects are mediated via the presynaptic CB_1_ receptor. CB_1_ receptor levels notably diminish at early stages of Huntington’s disease (HD) prior to the characteristic atrophy and neurodegeneration of medium spiny neurons (MSNs)^2^,^3^. In addition, the activation of CB_1_ receptor by Δ^9^-tetrahydrocannabinol (THC), the most abundant psychoactive compound of *Cannabis sativa*, exerts a disease attenuating action diminishing MSN neuronal loss^4^. Moreover, CB_1_ receptor signaling promotes neural progenitor cell proliferation and regulates neural cell differentiation^5^. Unfortunately the clinical implications for the neuroprotective cannabinoid actions are hampered by the psychoactive consequences of CB_1_ receptor signaling, which is the most abundantly expressed G-protein coupled receptor in the CNS. Therefore CB_1_ receptor targeting compounds exert undesired psychoactive actions by altering neurotransmitter release.

In addition to THC, more than 100 plant-derived compounds are known to exist, and for most of them their pharmacological profile is still far to be understood^6^. Among them, cannabidiol (CBD) and cannabigerol (CBG) constitute non-psychoactive cannabinoid compounds that do not bind to CB_1_ receptors and have also been tested as potential candidates for pharmacological therapies in HD patients and experimental models^7^,^8^. CBD alone or combined with THC (1:1), as formulated in sublingual spray Sativex (GW Pharmaceuticals) prevents neurodegeneration in animal models of toxin-induced striatal neurodegeneration^8^. These results prompted the development of a recent clinical trial to assess the safety of Sativex administration in HD patients (ClinicalTrials.gov, NCT01502046) that evidenced the lack of negative consequences of chronic Sativex administration^9^. More recently, we have found that CBG activated PPARγ in striatal cells thereby alleviating symptomatology and neuroinflammation in murine models of HD^7^.

Peroxisome proliferator-activated receptor-γ (PPARγ) is a nuclear receptor implicated in the regulation of lipid metabolism and glucose homeostasis and it is the target for glitazones, a class of oral antidiabetic drugs^10^. However, PPARγ is broadly expressed and has been recognized to play a key role in inflammatory processes and neurodegenerative diseases. In this sense, it has been shown that thiazolidinediones (TZDs) are neuroprotective in mutant huntingtin (mHtt) expressing cells and reduce mHtt aggregates in the brain^11^,^12^,^13^, thus supporting the concept that PPARγ may be a valid target for the management of HD. In addition, targeting PPARγ owing to its regulatory role of neural progenitor cell proliferation and differentiation^14^ constitutes a promising candidate to promote neural repair in neurodegenerative conditions.

TZDs, including the widely used drug rosiglitazone (RZG), are strong activators of PPARγ and have come under scrutiny because of their clinical side effects such as weigh gain, fluid retention, osteoporosis and other disorders^15^. However, more subtle modulation of PPARγ by small molecules such as cannabinoids may provide anti-inflammatory and neuroprotective activities resulting in more favourable outcomes.

In a step forward to improve the pharmacological profile and efficacy of natural cannabinoids different chemical modifications have been introduced and evaluated. For instance, quinone derivatives such as HU-331 and VCE-003 have been developed from CBD and CBG respectively^16^,^17^. HU-331 exerts a potent antitumoral activity by targeting DNA polymerase II, while VCE-003 has been identified as an immunosuppressant that exerts a protective action in models of multiple sclerosis (the Theiler’s murine encephalomyelitis virus and experimental autoimmune encephalomyelitis) through a PPARγ dependent pathway^16^,^18^. However, VCE-003 is a potential electrophilic compound that may complicate its development for chronic treatments. In this report we have developed a second generation of cannabigerol quinone derivatives such as VCE-003.2, which retain the ability to bind and activate PPARγ. VCE-003.2 is a non thiol-reactive compound with low adipogenic activity that shows neuroprotective and antiinflammatory activities in HD models *in vivo* and *in vitro*.

## Results

### VCE-003.2 is a non-thiol trapping derivative of CBG

**Figure 1:**
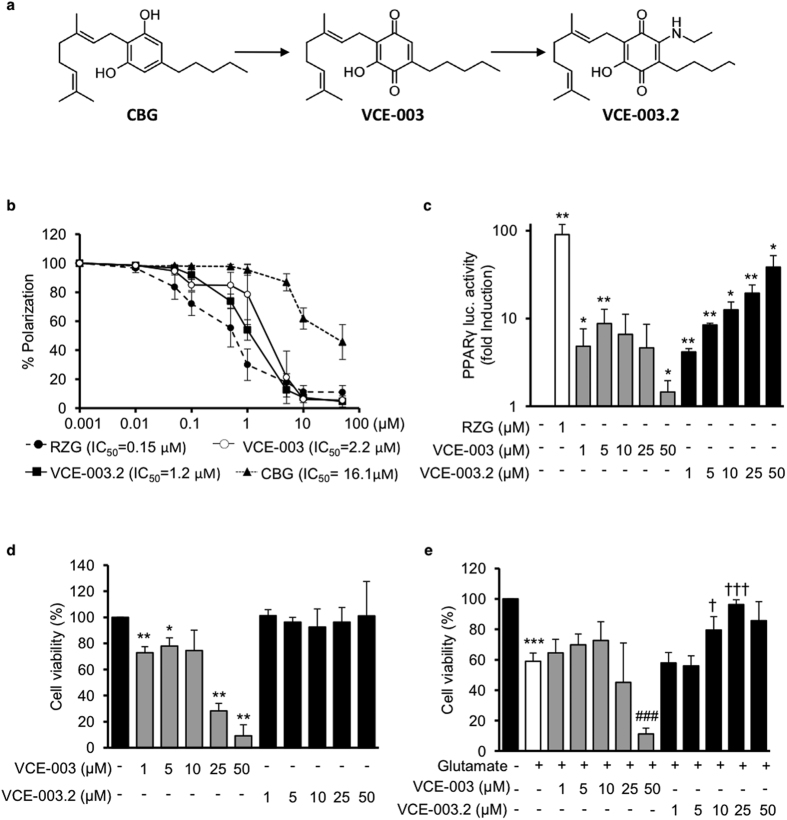
VCE-003.2 synthesis and characterization. (**a**) Schematic representation of VCE-003.2 synthesis. (**b**) PPARγ binding affinities of rosiglitazone (RZG), cannabigerol (CBG), VCE-003 and VCE-003.2 were measured by using a ligand competition assay. The indicated concentrations were tested and results were plotted to obtain the polynomial trend on a logarithmic range. IC_50_ values are indicated within the graph. (**c**) Effect of VCE-003 and VCE-003.2 on PPARγ transcriptional activity. HEK293 cells were co-transfected with GAL4-PPARγ and GAL4-luc and incubated with the indicated compound concentrations for 6 hours prior to luciferase activity determination. Results are expressed as the fold induction ± S.D. relative to untreated control. (**d**) N2a cells were incubated with VCE-003 and VCE-003.2 as indicated during 24 hours and cell viability was determined by the MTT assay. Results are expressed as percentage of cell viability against the negative control (100%). (**e**) N2a cells were pre-treated with the compounds for 30 min and incubated during 24 h with 15 mM glutamate and cell viability determined by MTT. Results are expressed as percentage of cell viability against the negative control (100%). All experiments are shown as mean ± S.D. from at least three independent experiments. Statistics: *^,#,†^p < 0.05, **^,##,††^p < 0.01 and ***^,###^p < 0.001.

We have previously shown that CBG oxidation to quinol (VCE-003) increases the PPARγ binding activity of this natural cannabinoid^16^. VCE-003 is also a potent immunosuppressor, which may be due to the electrophilic (thiol-trapping) nature of this quinone derivative^18^. In order to dissect the potential thiol-trapping activity of VCE-003 from the rest of its biological profile, the compound was subjected to C-H functionalization by the introduction of a nitrogen function as in VCE-003.2 (Fig. 1a). The Michael reactivity of VCE-003 and its analogue VCE-003.2 was investigated using a cysteamine recovery assay inspired by the cysteamine-trapping assay, an NMR method based on the treatment of a thiophylic compound with cysteamine in DMSO^19^. We found that VCE-003.2 can be recovered unscathed while in contrast VCE-003 was undetectable in the residue, indicating that it had irreversibly formed polar and not extractable adducts with cysteamine (Supplementary information). In addition, we found that VCE-003 induced reactive oxygen species (ROS) and activated the Nrf2 pathway, whereas none of these bioactivities were induced by VCE-003.2 (see Supplementary Fig. S1)

Next, we investigated whether VCE-003.2 was able to bind to PPARγ, and compared its binding capacity to VCE-003 and RZG. Using a PPARγ competitor-binding assay in 293T cells, we found that VCE-003.2 binds to the nuclear receptor with an IC_50_ of 1,2 μM that is in the range of affinity for VCE-003 (2.2 μM) (Fig. 1b). To further study the ability of VCE-003 and VCE-003.2 to activate PPARγ transcriptional activity, cells were transfected with a GAL4-PPARγ expression plasmid plus a GAL4-luc reporter plasmid. Our results showed that VCE-003 induced PPARγ transactivation in a biphasic manner and this activity was lost with the highest concentrations tested. On the contrary, VCE-003.2 induced PPARγ transactivation in a concentration-dependent manner (Fig. 1c). The results obtained with VCE-003 may reflect a cytotoxic activity and, therefore, we investigated the impact of VCE-003 and VCE-003.2 in cell viability. N2a cells were treated with increasing concentrations of both compounds and we found that VCE-003 but not VCE-003.2 showed a clear cytotoxic activity at the highest concentrations tested (Fig. 1d). Interestingly we found that VCE-003.2 was able to prevent excitotoxicity induced by glutamate in N2a cells (Fig. 1e). Altogether our results indicate that the specific modification introduced in the structure of VCE-003.2 did not affect its PPARγ binding activity but abolished its thiophilic and cytotoxic activity. We also investigated the binding profile of VCE003.2 to classic CB_1_ and CB_2_ receptors using ligand-binding assays. Our data confirmed that VCE003.2 has a poor binding affinity for both cannabinoid receptor types with predicted Ki >40 μM (data not shown).

### Effect of VCE-003.2 on adipogenic and osteoblastogenic differentiation

**Figure 2:**
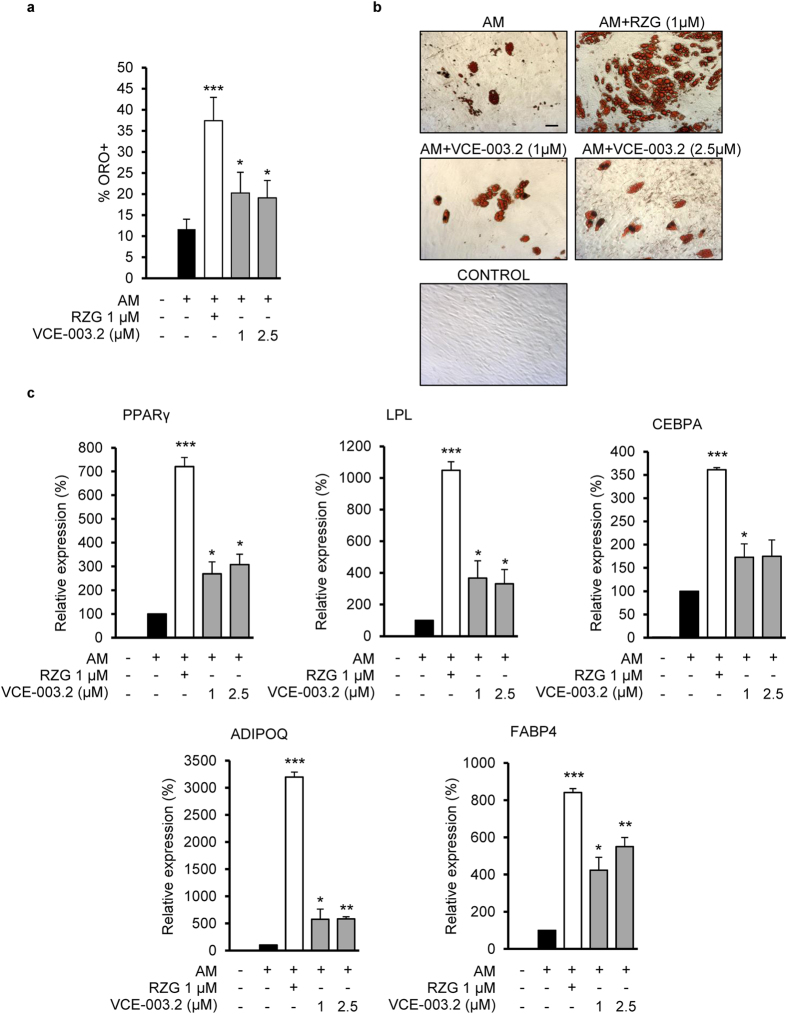
Effect of VCE-003.2 on adipogenic differentiation. Human mesenchymal stem cells (MSC) were differentiated in adipogenic medium (AM) in the presence of RZG or oil red O VCE-003.2 and adipogenic markers were characterized. (**a**) Quantification of oil red (ORO) positive cells after 21 days of differentiation. (**b**) Representative images of accumulated fat vesicles stained with oil red (Bar size, 50 μm). (**c**) Gene expression of PPAR-γ2, LPL, CEBPA, ADIPOQ and FABP4 in MSCs differentiated during 18 days. Data represent the percentage of increase over AM considered as the 100% of adipogenic induction. Experiments were performed from three different human MSC preparations each one obtained from a different bone marrow sample. Statistics: *p < 0.05, **p < 0.01 and ***p < 0.001 RZG or VCE-003.2 treatment vs. AM.

PPARγ is the target of TZDs that have been used extensively in patients with type 2 diabetes. However, these PPARγ activators, which are considered PPARγ full agonists (PPARγ-fa), have undesirable clinical side effects such are weight gain, peripheral edema, bone loss, and increased risk of hearth failure^14^. PPARγ also controls bone mass by regulating the differentiation of mesenchymal stem cells (MSCs) toward osteoblasts and adipocytes and it has been shown that TZDs suppresses osteoblasts and promotes adipocytes development^20^. However, the physiologic and therapeutic relevance of the PPARγ pathway have promoted new studies to develop newer classes of molecules that reduce or eliminate adverse effects. Therefore, much progress has been achieved in the discovery and development of selective PPARγ modulators (PPARγ-m) as safer alternatives to PPARγ-fa^21^. Therefore, the ability of VCE-003.2 to influence MSCs differentiation into osteoblasts and adipocytes was monitored in adipogenic medium (AM) and osteoblastogenic medium (OM) during 21 days of differentiation. The capacity of both media to induce differentiation into these cellular types has been proved and described previously^22^. The effect of VCE-003.2 on adipocyte differentiation was tested at 1 and 2.5 μM and compared to the effect of 1 μM RZG. As depicted in Fig. 2a VCE-003.2 never achieved the percentage of Oil Red O positive cells (ORO^+^) induced by RZG. Morphological examination showed that lipid droplets are less numerous and smaller in cells treated with VCE-003.2 compared to RZG (Fig. 2b). Consistent with a weaker adipogenic activity *in vitro*, the expression levels of adipogenic differentiation markers such as PPARγ, LPL, CEBPA, ADIPOQ and FABP4 were sensibly lower in cells treated with VCE-003.2 compared to cells treated with RZG (Fig. 2c). It has been shown that full PPARγ agonists such as glitazones also suppress MSC osteoblast development^23^, which could explain bone loss after prolonged used of this class of drugs^24^. Interestingly, we found that VCE-003.2 did not inhibit the expression of osteogenic differentiation markers such as Runx2, SP7, ALP and IBSP (Supplementary Fig. S2). In summary we show that VCE-003.2 qualifies as a PPARγ-m since it is significantly less adipogenic than RZG and does interfere with osteoblasts differentiation.

### VCE-003.2 exerts a prosurvival action in neural progenitor cells

**Figure 3:**
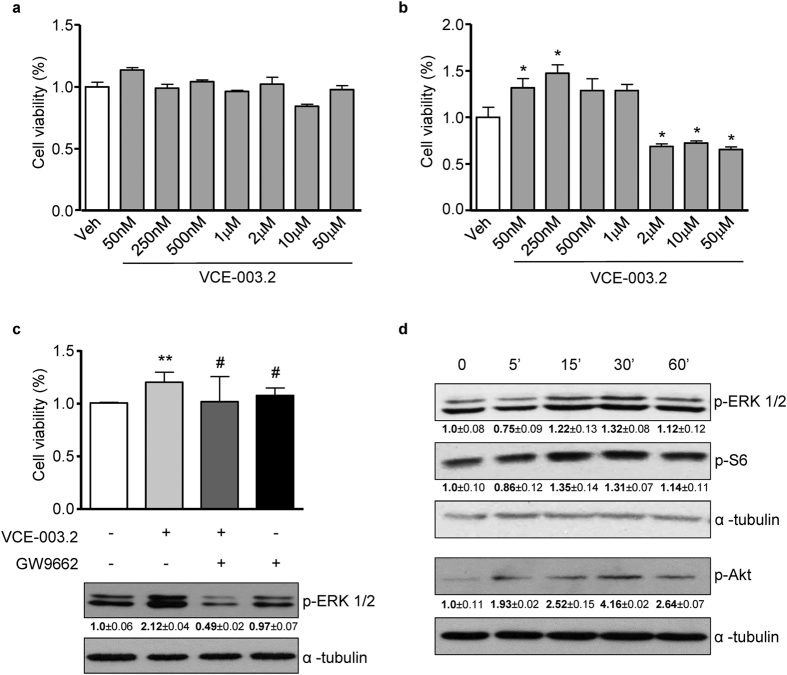
Prosurvival effect of VCE-003.2 in hippocampal neural progenitor HiB5 cells. (**a**,**b**) HiB5 cells in proliferative and differentiation conditions respectively were incubated with increasing concentrations of VCE-003.2 and cell viability was assessed using the MTT test after 48 h. (**c**) Cell viability and ERK_1/2_ phosphorylation status (activation) was determined in VCE-003.2-treated cells in the absence or presence of GW9662 (5 μM). (**d**) VCE-003.2 time-dependent regulation of the ERK, Akt and mTORC1 signaling pathways were determined by incubation with anti-phospho-ERK, phospho-Akt and phospho-S6 antibodies. Loading control was performed with α-tubulin antibody. Representative western blots and densitometry quantification of three independent experiments are shown. Values are expressed as means ± S.D. *p < 0.05; **p < 0.01 vs. Vehicle; ^#^p < 0.05 vs. VCE-003.2.

To investigate the effect of VCE-003.2 in neural progenitor (NP) cells the HiB5 cell line was employed. HiB5 cells grown under proliferative conditions were exposed to increasing concentrations of the compound (50 nM to 50 μM). Cell viability assays did not reveal any effect indicating that VCE-003.2 is not toxic even at the highest concentrations employed (Fig. 3a). VCE-003.2 action was also determined during neuronal differentiation. In these conditions, VCE-003.2 exerted a prosurvival activity at lower concentrations, while at high concentrations it was toxic and reduced cell viability (Fig. 3b). Increased cell survival correlated with higher number of cycling cells revealed by immunofluorescence for the proliferating cell nuclear antigen (data not shown).

Next, we analyzed whether PPARγ was responsible for the prosurvival activity of VCE-003.2. Blockade of PPARγ with the antagonist GW9662 (5 μM) prevented VCE-003.2 (250 nM) action. Interestingly, the prosurvival activity of VCE-003.2 paralleled with the induction of extracellular signal-regulated kinase (ERK) phosphorylation, which was also abolished in the presence of GW9662 (Fig. 3c). Moreover, the activation status of ERK, Akt and mammalian target of rapamycin complex 1 (mTORC1) pathways typically associated to phytocannabinoid-induced neural cell fate regulatory actions^5^ was determined in time course experiments with VCE-003.2 using specific antibodies for the phosphorylated and activated forms of ERK, Akt, and the mTORC1 target the S6 protein (Fig. 3d). VCE-003.2 induced a transient activation of ERK and mTORC1 signaling pathways between 15 and 30 min after stimulation. In addition, Akt was also activated at a higher extent and shortly after VCE-003.2 incubation and lasted until 60 min after incubation. Overall these results reveal a prosurvival action of VCE-003.2 in differentiating NPs that can be mediated through the PPARγ pathway.

### Neuroprotective activity of VCE-003.2 in Huntington-like induced neurodegeneration *in vitro*

**Figure 4:**
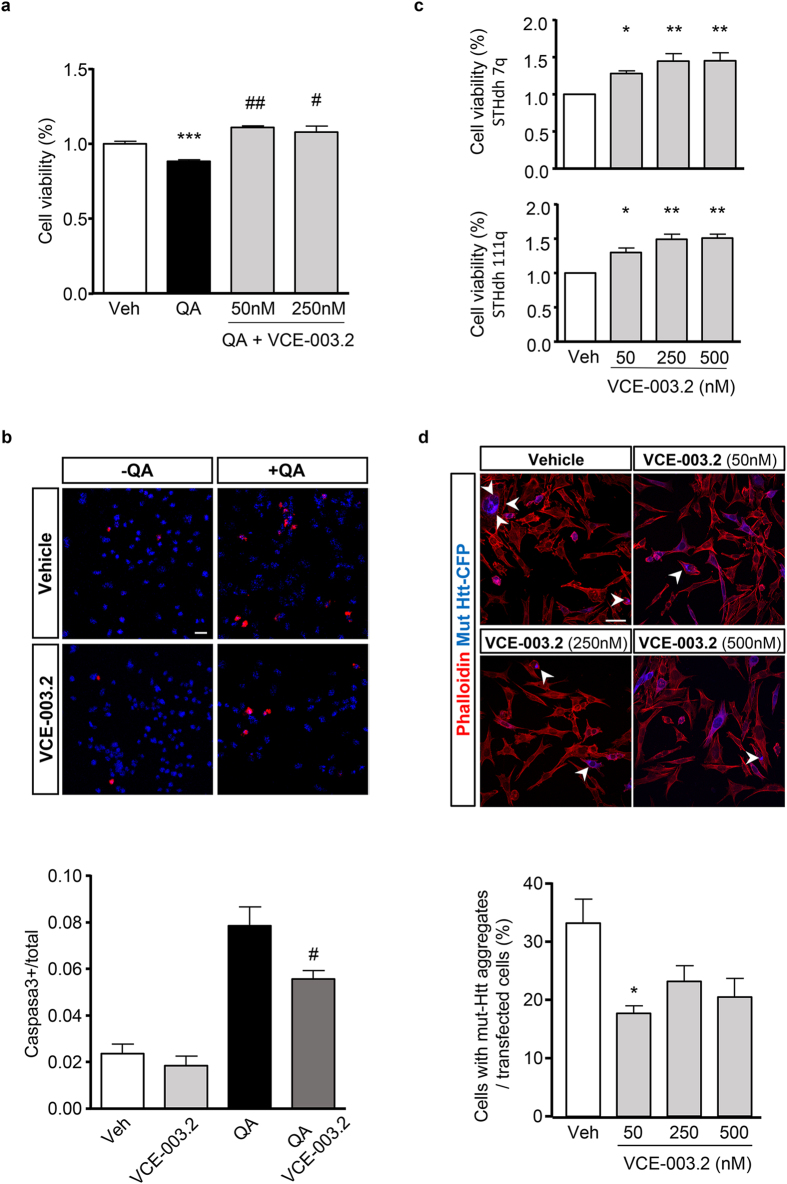
*In vitro* neuroprotective action of VCE-003.2. (**a**) HiB5 cells were treated with quinolinic acid (QA; 2,5 mM) in the presence or the absence of VCE-003.2 (50 and 250 nM) and cell viability was determined after 24 h by the MTT assay. (**b**) QA-induced apoptosis measured by cleaved caspase 3 immunostaining (upper panel) of differentiating HiB5 cells was quantified in the presence or absence of VCE-003.2 (250 nM). Cell counts were referred to total cell nuclei counterstained with DAPI and c-caspase3^+^ cell quantification is shown in the lower panel. (**c**) STHdh^7Q/7Q^ and STHdh ^111Q/111Q^ were serum deprived and incubated with the indicated VCE-003.2 concentrations for 72 h. Neuronal survival in the presence of VCE-003.2 was determined by MTT and referred to vehicle-treated neurons. (**d**) Aggregation of mut-Htt was assessed by fluorescence microscopy in immortalized striatal STHdh transfected cells with a CFP-tagged expression vector of mutant huntingtin (mut-Htt) and counterstained with fluorescent phalloidin (upper panel). Cells with mut-Htt aggregates with the indicated concentrations of VCE-003.2 were quantified and referred to total transfected cells (lower panel). Results are representative of three independent experiments. Values are expressed as means ± S. D. *p < 0.05 and ***p < 0.001 vs. Vehicle; ^#^p < 0.05 ^##^p < 0.01 vs. QA. Bar size, 25 μm (4b) and 50 μm (4c).

Excitotoxicity is a hallmark of neurodegeneration including Huntington’s disease and thus quinolonic acid (QA) administration constitutes a widely employed model to investigate the mechanisms of striatal neurodegeneration. Since VCE-003.2 prevented excitotoxicity induced by glutamate (Fig. 1e), we also investigated the action of VCE-003.2 *in vitro* after the excitotoxicity insult evoked by QA treatment in HiB5 cells. We show that QA-induced NP cell death was fully prevented by the coincubation with VCE-003.2 (Fig. 4a). In addition, QA-induced apoptosis measured by immunofluorescence against cleaved caspase 3 was also significantly inhibited in the presence of VCE-003.2 (Fig. 4b). Next we analyzed the impact of VCE-003.2 in immortalized striatal neuroblasts expressing full-length huntingtin with 7 glutamines (STHdh^Q7/Q7^) or mutant huntingtin bearing 111 glutamines in the N-terminal domain (STHdh^Q111/Q111^). Importantly, neuronal viability after serum deprivation was improved by VCE-003.2 in both STHdh^Q7/Q7^ and STHdh^Q111/Q111^ (Fig. 4c). We also investigated if VCE-003.2 could also interfere with mutant huntingtin (mut-Htt) aggregation. Striatal STHdh cells were transfected with exon 1 mut-htt expression vector encoding 94 expanded polyglutamine repeats. While in vehicle-treated cells mut-htt formed protein aggregates in numerous neurons, VCE-003.2 treatment reduced the number of cells with aggregates (Fig. 4d). These findings demonstrate that the prosurvival action of VCE-003.2 in differentiating NPs is also translated to *in vitro* models of striatal neurodegeneration.

### Neuroprotective effects of VCE-003.2 in murine models of Huntington’s disease

**Figure 5:**
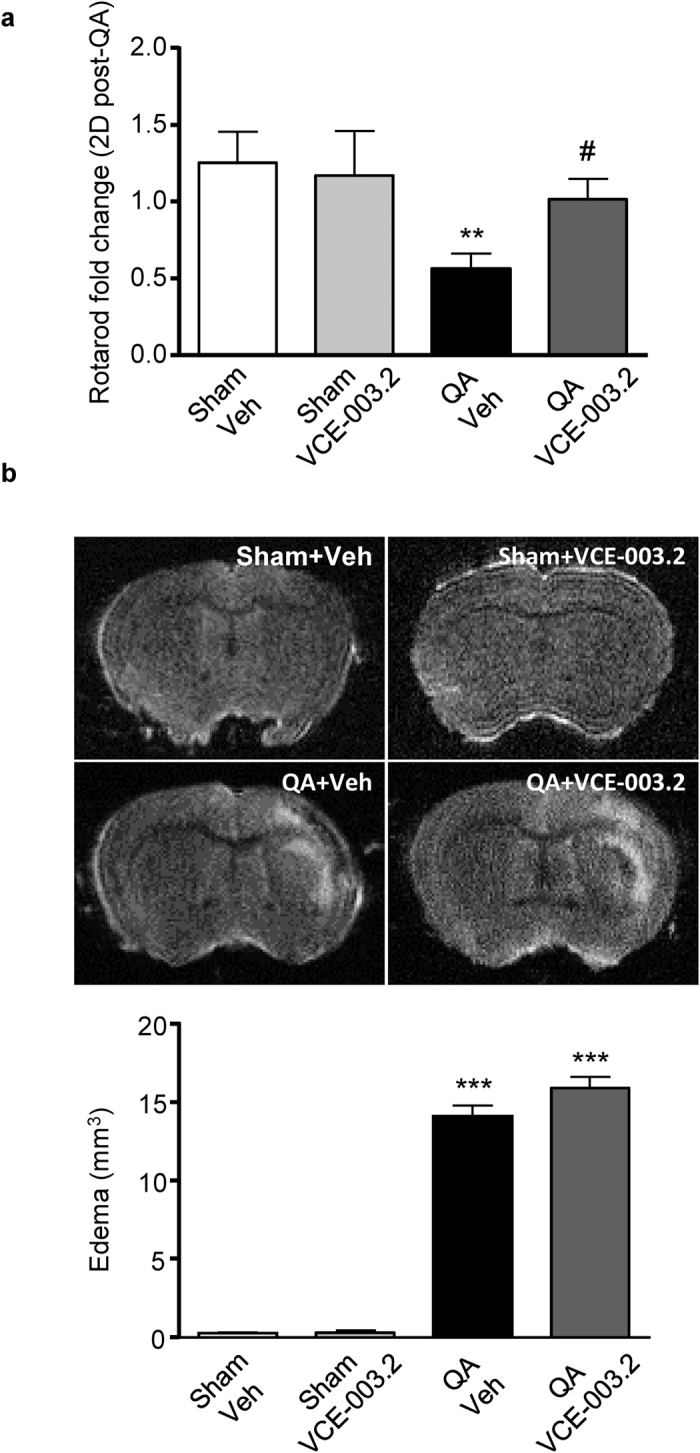
Protective action of VCE-003.2 administration in QA-induced excitotoxicity *in vivo*. (**a**) CD1 mice were subjected to quinolinic acid (QA) intrastriatal injection and RotaRod performance (fold change) was determined 2 days after lesion in vehicle- and VCE-003.2-treated mice (10 mg/kg). (**b**) Representative MRI images (Upper panel) and edema volume quantification (lower panel) 5 days after QA injection. Values are expressed as means ± SEM. Sham-Veh and Sham-VCE (n = 7 in each group); QA-Veh (n = 8); QA-VCE (n = 7). **p < 0.01, ***p < 0.001 vs. Vehicle. ^#^p < 0.05 vs. QA.

**Figure 6:**
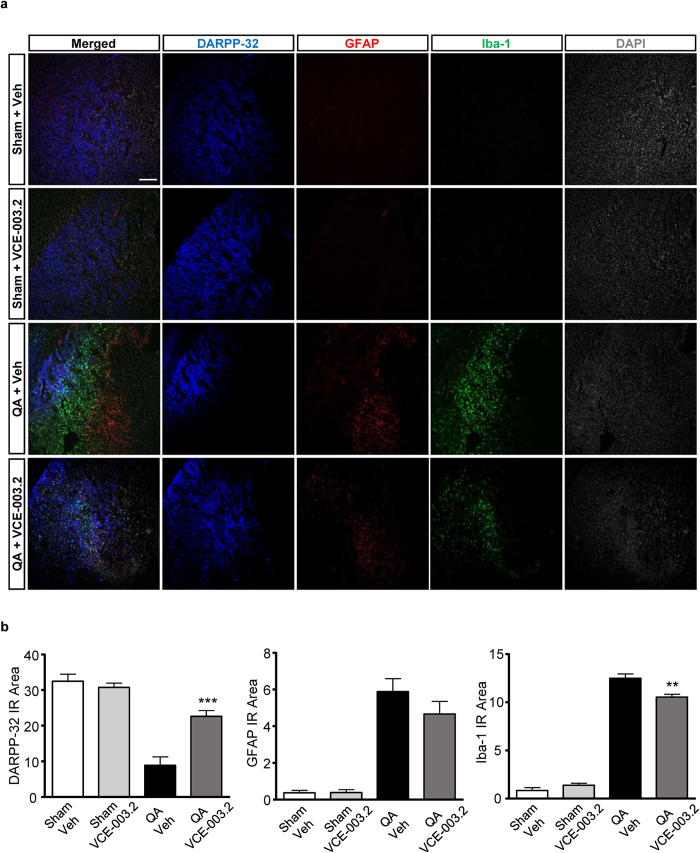
Neuroprotection and attenuation of glial reactivity by VCE-003.2 in QA-induced excitotoxicity *in vivo*. (**a**) Representative immunofluorescence images of DARPP32, GFAP, Iba-1 are shown in sham and QA-injected mice treated with vehicle or VCE-003.2 (10 mg/kg). (**b**) Immunoreactivity of the different markers was quantified with Image J software and referred to the striatal surface analyzed. Values are expressed as means ± SEM. Sham-Veh and Sham-VCE (n = 3); QA-Veh (n = 8); QA-VCE (n = 6). **p < 0.01, ***p < 0.001 vs. QA. Bar size, 200 μm.

To assess the pathophysiological relevance of the neuroprotective action of VCE-003.2 *in vivo,* we firstly employed the intrastriatal QA-induced model of Huntington’s disease. The functional impact of striatal excitotoxicity was analyzed by quantification of the RotaRod test performance. Administration of QA induced a decline in RotaRod performance 2 days after injury that was reversed by VCE-003.2 (Fig. 5a). Similar results were found 1 and 3 days after injury (Supplementary Fig. 3). MRI analyses were employed to quantify brain edema. However, despite improved motor function in VCE-003.2-treated mice edema volume was not affected 5 days after injury (Fig. 5b). Next, we sought to investigate if VCE-003.2 exerted any positive action in striatal neurodegeneration and gliosis. At the cellular level, VCE-003.2 prevented QA-induced DARRP32 neuronal loss and microglial activation, and also revealed a tendency to attenuate reactive astrogliosis (Fig. 6).

**Figure 7:**
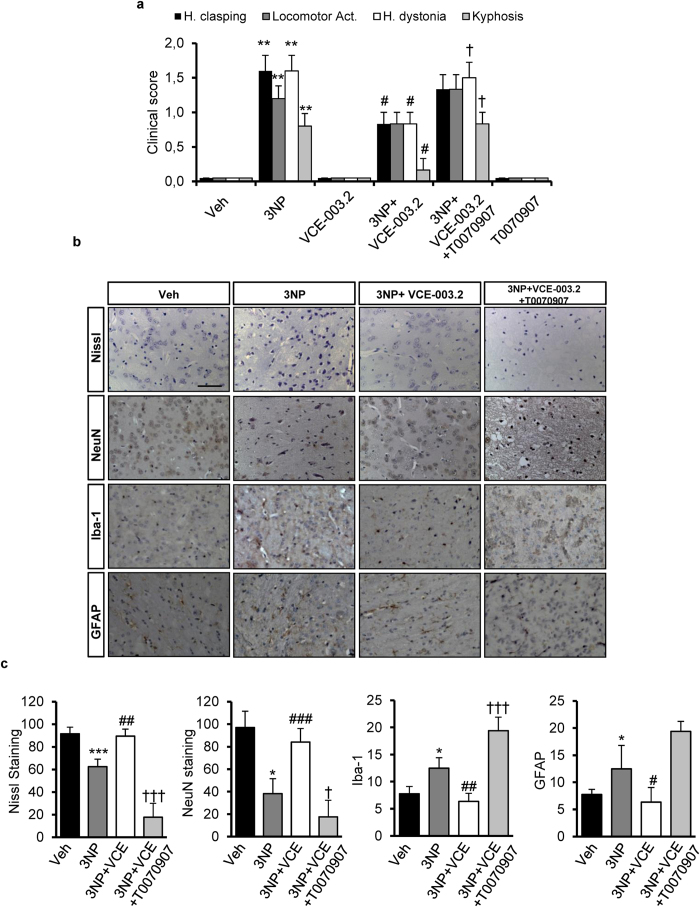
VCE-003.2 is neuroprotective in 3NP-intoxicated mice. (**a**) Behavioural score was determined 12 hours after 3NP intoxication. Mice were treated with VCE-003.2 (20 mg/Kg) alone or combined with T0070907 (5 mg/kg) as indicated. Animals were also treated separately with either VCE-003.2 or T0070907 alone. Hind limb clasping, general locomotor activity, hind limb dystonia and kyphosis were rated from 0 to 2 based on severity (score 0 indicates normal function and 2 indicates the most severe affected function). Values are expressed as means ± SEM (n = 6). (**b**) Representative images of Nissl staining performed on coronal striatal brain sections from the same mice groups. Photomicrographs of NeuN (Bar size, 50 μm) Iba-1 and glial fibrillary acidic protein (GFAP) immunostained sections through the different group of mice are also shown. (**c**) Quantification of the different markers was performed with Image J software. Total average number of neurons (NeuN^+^), microglia (Iba1^+^) and astrocytes (GFAP^+^) is shown. Values are expressed as means ± SEM (n = 6). *p < 0.05, **p < 0.01, ***p < 0.001 vs. Vehicle; ^#^p < 0.05, ^##^p < 0.01, ^###^p < 0.001 vs. 3NP; †p < 0.05, ^††^p < 0.01, ^†††^p < 0.001 vs. the 3NP + VCE-003.2 group.

In order to confirm the neuroprotective action of VCE-003.2 we used a second model of Huntington’s disease based on 3-nitropropionic acid (3NP) administration. Treatment with 3NP results in several alterations including neurological, histological and biochemical changes characteristic of some aspects of HD pathology. 3NP-treated mice exhibited high scores in hindlimb clasping, locomotor activity, hindlimb dystonia and kyphosis compared with control animals treated with vehicle (Fig. 7a). Treatment with VCE-003.2 improved the motor deficits of 3NP-lesioned mice by reducing hindlimb clasping, dystonia and kyphosis. A similar tendency was observed for locomotor activity although it was not statistically significant (Fig. 7a). In contrast, when mice were co-administered with the PPARγ antagonist T0070907 the effect of VCE003.2 was abrogated. Neither VCE-003.2 nor T0070907 alone influenced the behavior of the animals.

We next investigated the expression of specific proinflammatory markers. In 3NP-lesioned mice we observed an upregulation of mRNA levels of the inflammatory enzyme cyclooxygenase-2 (COX-2) and the proinflammatory cytokines TNF-α and IL-6 (Table 1). Treatment with VCE-003.2 inhibited the upregulation of these proinflammatory markers induced by 3NP, and coadministration of the PPARγ antagonist prevented the anti-inflammatory action of VCE-003.2. We also investigated the impact of VCE-003.2 in striatal degeneration and atrophy. The administration of 3NP reduced the number of neurons in the striatum, as determined by Nissl staining and NeuN immunohistochemistry (Fig. 7b,c). Notably, VCE-003.2 administration exerted a neuroprotective action and rescued the loss of Nissl and NeuN^+^-cells in 3NP lesioned mice and co-administration of T0070907 prevented the activity of VCE-003.2. In addition, VCE-003.2-mediated neuroprotection was associated with reduced 3NP-induced microgliosis and astrogliosis as determined by Iba1 and GFAP immunohistochemistry (Fig. 7b,c). Importantly, not only did VCE-003.2 reduce the neuroinflammatory burden of striatal neurodegeneration, but it also improved different oxidative stress markers (Table 1), thus increasing catalase activity, reduced glutathione (GSH) levels, and superoxide dismutase (SOD) activity.

It is interesting to note that T0070907 did not prevent the effect of VCE-003.2 on catalase activity and GSH expression suggesting that VCE-003.2 also exert PPARγ-independent activities. In this sense it has been reported that classical PPARγ agonists also showed antioxidant and anti-inflammatory activities through PPARγ-independent pathways^25^,^26^. Although T0070907 alone did not induce the expression of COX-2 and IL-6 mRNAs in the brain in combination with 3-NP these markers were significantly upregulated (Table 1). We do not know why T0070907 enhances specifically the expression of COX-2 and IL-6 in 3-NP-intoxicated mice but this result is not without precedents as GW9662, another irreversible PPARγ inhibitor, was toxic for dopaminergic neurons in MPTP-treated mice^25^.

## Discussion

The development of cannabinoid quinonoid compounds displaying neuroprotective and anti-inflammatory activities, which are non-cytotoxic and more specific in their actions, is a major therapeutic goal. Therefore in the present study we aimed to identify new plant-derived molecules with better pharmacological properties facilitating the assessment of their neuroprotective potential in Huntington’s disease-associated neurodegeneration. We have identified VCE-003.2 as a novel CBG-quinone derivative compound which, acting via PPARγ activation, exerts a neuroprotective activity thus attenuating neuronal death *in vivo* and *in vitro* and promoting neuronal progenitor survival. VCE-003.2 outperforms CBG to bind and transactivate the nuclear receptor PPARγ and compared to RZG, a potent PPARγ full agonist, VCE-003.2 does not interfere with osteoblast differentiation and is less adipogenic. Thus, this novel compound may qualify as a selective and safe PPARγ modulator based on cannabinoid structural motif. Noteworthy, VCE-003.2 activates prosurvival signalling pathways that not only contribute to its neuroprotective action *in vivo* and *in vitro*, but also promote neural progenitor survival during differentiation. Importantly, while some phytocannabinoids are considered safe compounds devoid of deleterious actions^6^ and can exert a pro-neurogenic response^27^,^28^, this may not always be the case. For instance, CBD, a promising non-psychotropic compound of therapeutic value^6^, increases the sensitivity to oxidative stress damage of differentiating neurons^29^. Our new findings demonstrate the possibility that improved phytocannabinoid-derived drugs can be oriented by chemical modification to improve their safety profile and promote the endogenous neurogenic response induced upon neuronal loss^30^.

The utility of electrophilic compounds as anti-cancer drugs is unquestioned, as demonstrated by FDA-approved compounds such as doxorubicin, mitomycin C, and mitoxantrone. However, the use of this class of drugs for chronic treatments is not feasible because of its reactivity and toxicity. The electrophilic and redox-cycling properties of electrophiles appear to induce cytotoxicity either by covalent alkylation of DNA^31^,^32^ or through induction of cellular reactive oxygen species (ROS)^33^,^34^. For example, doxorubicin, a quinone-containing compound, predominantly causes cytotoxicity by depletion of GSH *in vitro* and *in vivo*^35^. However some electrophiles of the type *ortho* and *para*-quinones, among others, can be also neuroprotective by inducing an electrophilic counterattack, a system that detoxifies electrophiles and removes them immediately^36^. Electrophilic counterattack by mild pro-oxidants that are non-cytotoxic is mediated by the activation of the Keap1/Nrf2 and HSP90/HSF-1 transcriptional pathways, which provide an effective redox regulation by activating cellular antioxidant genes and by restoring GSH levels in cells^37^,^38^. Accordingly, hydroquinone and catechol by generating the corresponding quinones activate both pathways and protect neuronal cells against excitotoxicity induced by glutamate that induced cytotoxicity via GSH depletion^39^. Our data showing that VCE-003.2 prevented glutamate-induced cytotoxicity in N2a cells and restored *in vivo* the levels of GSH decreased by 3NP intoxication suggest that this compound is a non-cytotoxic quinone that favours electrophilic counterattack.

Interestingly, the resorcinol moitety of CBD is converted to CBD-hydroxy-quinone during metabolism with mouse hepatic microsomes^40^, and we have found that CBD-hydroxy-quinone activates the Keap1/Nrf2 pathway (unpublished results). Thus, CBD quinone metabolites may partly explain the antioxidant activity of this cannabinoid *in vivo*. Whether CBG is also converted to CBG-hydroxy-quinone (precursor of VCE-003.2) during liver metabolism remains to be investigated. However CBG, which is not an antioxidant molecule, also restores the levels of GSH in the brain of 3NP-lesioned mice^7^.

Plant-derived phytocannabinoid administration and activation of their canonical G-protein coupled cannabinoid receptors have already been shown to exert a neuroprotective action in striatal degeneration models of Huntington’s disease. THC administration exerts a neuroprotective effect on R6/2 transgenic and toxin-based models of Huntington’s disease via CB_1_ receptor signaling^4^. CB_1_ receptors located in corticostriatal projection neurons constitute the particular subpopulation of neuroprotective receptors by engaging a PI3K/mTORC1-mediated production of BDNF^41^. However, in other transgenic models, such as R6/1 in which striatal neurodegeneration and symptoms appear in a much more extended time, 8-week treatment with different cannabinoid ligands or endocannabinoid raising compounds did not improve the pathology progression^42^. Taking into account that HD progression occurs concomitantly with a very early decline of presynaptic CB_1_ receptors^2^,^4^, the use of pure CB_1_ agonists alone may have a limited therapeutic window. Thus, the current scenario indicates that targeting CB_1_ receptors may be a plausible therapeutic strategy in the initial stages of HD, but later this shall be replaced by the use of alternative drugs, that is, with anti-inflammatory effects. In this regard, drugs targeting CB_2_ cannabinoid receptors or PPARγ, the nuclear receptor for some cannabinoids, have been shown to be beneficial by attenuating either microglial/macrophage innate immunity or peripheral adaptative immune infiltration^43^,^44^. Thus, in HD animal models, the administration of PPARγ agonists protects mut-Htt-induced striatal neurodegeneration, attenuates neuroinflammation and decreases oxidative damage^11^,^12^,^13^. Evidence accumulated over the past few years have implicated an impaired function of PPAR-γ coactivator-1α (PGC-1α), a transcriptional master coregulator of mitochondrial biogenesis and cellular metabolism, in causing mitochondrial dysfunction in HD. In this sense PPARγ agonists have beneficial effects on mitochondrial dysfunction, thus contributing to the pathogenesis of HD^45^. We are currently investigating the effect of VCE-003.2 on mitochondria biogenesis *in vitro* and in genetic models of HD *in vivo*.

The therapeutic potential of the novel plant-derived cannabinoid compound characterized herein can overcome some of the therapeutic hurdles faced by cannabinoid-based drugs in neurodegenerative diseases. Great expectations exist in regard of the therapeutic uses of cannabinoid compounds as symptomatic relief drugs, as already demonstrated and approved for neuropathic pain and spasticity in multiple sclerosis^1^. Overall, the emerging scenario of cannabinoid-based therapies in basal ganglia and other neurodegenerative disorders suggests that the use of specific cannabinoid preparations with different molecular targets and chemical improvement of their pharmacological properties can achieve maximal benefits attenuating disease progression and symptoms.

## Methods

### Cell lines and reagents

The HiB5 hippocampal progenitor cell line (kindly provided by Prof. Z. Kokaia, Lund Stem Cell Center, Sweden) was grown as described^46^ in DMEM supplemented with 2 mM glutamine, 1% penicillin/streptomycin and 10% (v/v) fetal calf serum (FCS; Lonza, Switzerland). HiB5 cell cultures were incubated in 5% CO2 at 33 °C, the proliferation-permissive temperature of the oncogenic tsA58 allele of the SV40 large T antigen. Incubation at 37 °C loss of proliferative capacity and neural differentiation. Cell viability was determined after 24 h using the 3-[4,5-dimethylthiazol-2-yl]-2,5-diphenyltetrazolium bromide (MTT) tesresults int. Immortalized mouse striatal STHdh cells (kindly provided by S. Gines, U. Barcelona, Spain) were cultured as described^47^. Cell were transiently transfected with 10 μg doxycycline-inducible CFP-huntingtin-94Q using Amaxa´s Nucleofector following the instructions of the manufacturer (Lonza). In addition STHdh^Q7/Q7^ and STHdh^Q111/Q111^ expressing endogenous levels of full-length huntingtin with 7 glutamines or 111 glutamines in the protein N-terminal domain, respectively, were used. The HEK293 and the neuroblastoma Neuro-2a (N2a) cell lines (ATTC, Germany) were growth in DMEM supplemented with 10% FCS and antibiotics. Rosiglitazone (RZG), T0070907 and GW9662 were purchased from Cayman Chemical Company (Ann Arbor, MI, USA). Cannabigerol (CBG) was from THC Pharm (Frankfurt am Main, Germany). All other reagents were from Sigma-Aldrich (St. Louis, MO, USA) unless indicated. The synthesis and structure of VCE-003 was previously published^16^ and for VCE-003.2 it is described in Supplementary Information.

### PPARγ binding and transcriptional assays

To determine PPARγ binding activity the PolarScreen^TM^ PPAR Competitor Assay kit (Life Technologies, CA, USA) was used following the manufacturer’s instructions. The IC_50_ value was determined as the 50% inhibition of percentage of polarization. To study PPARγ transcriptional activity HEK293 cells were seeded in 24-well plates and transiently co-transfected with the expression vector GAL4-PPARγ and the luciferase reporter vector GAL4-luc using Roti-Fect (Carl Roth, Karlsruhe, Germany) following the manufacturer’s instructions. To correct for transfection efficacy, 100 ng *Renilla* luciferase (pRL-CMV) was also cotransfected. After stimulation, the luciferase activities were quantified using Dual-Luciferase Assay (Promega, Madison, WI, USA).

### Cytotoxicity assays

Briefly, N2a cells were seeded at a density of 1 × 10^3 ^cells/well in 96-well plates, treated with increasing concentrations of VCE-003 or VCE-003.2 for 24 hours and cell viability was measured by the MTT method. For neuroprotective assays N2a cells were pre-incubated for 1h with either VCE-003 or VCE-003.2, as indicated, and then treated with 15mM glutamate during 24 hours. Control cells were set as 100% and data were referred to that value.

### Human mesenchymal stem cells (MSCs) differentiation

Reina Sofia University Hospital review board. The Reina Sofia University Hospital Review Board approved this study and the procedures followed were in accordance with the ethical standards of the ethic committee from Hospital Reina Sofía and with the Declaration of Helsinki. Bone marrow donors were recruited by the Haematology Service. All subjects gave their informed consent so that the bone marrow aliquots extracted for clinical purposes could also be used for mesenchymal bone marrow research. MSCs derived from bone marrow were seeded in α- MEM containing, 15% FCS, 2 mM UltraGlutamine, 1 ng/ml bFGF and antibiotics. Adipogenic and osteoblast differentiation was performed as described^22^. Treatment with RZG and VCE-003.2 started at the same time as the differentiation process. To confirm adipogenesis, cells that had been cultured in adipogenic media (AM) during 21 days were stained using oil red O. Cells were fixed in 3.7% paraformaldehyde for 10 min, washed with H_2_O, and incubated with an oil red O solution for 20 min at room temperature (RT). The images captured with the light microscope were analyzed with the ImageJ Software (NIH; Bethesda, MD, USA).

### Gene expression

Cells were collected at day 14 of differentiation and total RNA was extracted using the High Pure RNA Isolation kit (Roche Diagnostics). The total amount of RNA extracted was quantified using NanoDrop 2000 (Thermo Fisher Scientific Inc). Total RNA (1 μg) was retrotranscribed using the iScriptTM cDNA Synthesis Kit (Bio-Rad; Hercules, CA, USA), and the cDNA generated was analyzed by real-time PCR, using the iQ^TM^ SYBR Green Supermix (Bio-Rad; Hercules, CA, USA). Real-time PCR to quantify the mRNA of osteoblastic and adipocityc markers was performed using a CFX96 Real-time PCR Detection System (Bio-Rad; Hercules, CA, USA). The HPRT gene was used to standarize mRNA expression in each sample. Gene expression was quantified using the 2-ΔΔCt method and the percentage of relative expression against the differentiated control (AM or OM) was represented. The primers used in this study are described in supplementary material.

### Western blot

Cleared cell extracts were subjected to SDS-PAGE, transferred to polyvinylidene difluoride membranes, and incubated with the correspondent primary antibodies against phospho-ERK1/2 (Thr202/Tyr204; Cell Signaling Technology, Danvers, MA, USA), phospho-S6 ribosomal protein (Ser235/236) (Cell Signaling Technology), phospho-Akt (Ser473) (Cell Signaling Technology) and α-tubulin (Sigma-Aldrich) overnight at 4 °C. After washing membranes, horseradish peroxidase-conjugated secondary antibody was added and detected by chemiluminescence system. Loading controls were performed with an anti-α-tubulin antibody. Densitometric quantification of the luminograms was performed using a GS-700 Imaging densitometer (Bio-Rad, Hercules, CA) and Image J software.

### *In vivo* models of neurodegeneration

Mice were housed under standard conditions (12-h light/dark cycle) in groups with access to food and water *ad libitum*. All experiments were performed in accordance with European Union guideline and approved by the Animal Research Ethic Committee of Córdoba University (2014PI/017) (3NP model) and by the Animal Research Ethic Committee of Complutense University (CEA-UCM 73/2012) (QA model). Procedures were designed to minimize the number of animals used and their suffering. Excitotoxicity was induced by intrastriatal quinolinic acid (QA) administration (100 nmol in 1 μL PBS solution, unilaterally) as previously described^41^ at the following coordinates: +0.6 posterior to bregma, +1.85 medio-lateral and -2.7 dorso-ventral to dura, in 12-week-old CD1 male mice (Harlan Ibérica, Barcelona, Spain). Pharmacological manipulation with VCE-003.2 (10 mg/kg body weight) was performed i.p. daily in 2% Tween 20 and 3% DMSO in saline buffer until sacrifice. Control animals received the corresponding vehicle injections. Motor function was assessed by RotaRod analysis conducted with acceleration from 4 to 40 rpm over a period of 570 s in an LE8200 device (Harvard Apparatus, Barcelona, Spain). Striatal neurodegeneration was also induced in 16-week-old C57BL/6 male mice (Harlan Ibérica, Barcelona, Spain) by seven intraperitoneal (i.p.) injections of 3-nitropropionic acid (3NP) (50 mg/kg; one injection each every 12 h prepared in PBS). 3NP-treated animals and their respective non-lesioned controls (injected with PBS) were used for pharmacological studies with VCE-003.2. Treatments consisted of 4 i.p. injections every 24 h with 20 mg/kg VCE-003.2 or vehicle (4% DMSO plus 6,2% Tween 20 in saline buffer), with the first and the last injections 30 min before the first and the last injections of 3NP, respectively. In some experiments, mice received a combination of VCE-003.2 and the PPARγ antagonist T0070907 (5 mg/kg) injected 15 minutes before VCE-003.2. In 3NP-lesioned mice, motor activity, hindlimb clasping, dystonia, and truncal dystonia were evaluated following previously described procedures^48^. All behavioural tests were conducted prior to drug injections to avoid acute effects of the compounds under investigation and all animals were euthanized 12 h after the last injection of 3NP and their brains removed. The right hemispheres were used to dissect the striatum which was frozen in RNAlater (Sigma-Aldrich) and stored at −80 °C for biochemical analyses. The left hemispheres were fixed in fresh 4% paraformaldehyde (in 0.1 M PBS) for 48 h at 4 °C and embedded in paraffin wax for staining and immunohistochemical analysis. In all experiments, at least 6 animals were used per experimental group.

### Magnetic resonance imaging

MRI was performed in excitotoxicity experiments the day before sacrifice at the Nuclear Magnetic Resonance Center of Complutense University (Madrid, Spain) using a Biospec 47/40 (Bruker, Ettlingen, Germany) operating at 4.7 Teslas, equipped with a 12 cm gradient set and using a 4 cm radio frequency surface coil. 3D T2-weighted spin-echo images were acquired using a fast spin-echo sequence. Diffusion water images delineated the area of neuroinflammation evidenced as hyperintense signals were quantified.

### Immunohistochemistry and confocal microscopy

Free floating coronal brain slices (30 mm) were processed as previously described^41^. In brief, after blocked with 10% goat serum, brain sections after were incubated with the indicated primary antibodies followed by secondary antibody incubation (2 hours at room temperature). The appropriate mouse, rat and rabbit highly cross-adsorbed AlexaFluor 488, AlexaFluor 594 and AlexaFluor 647 secondary antibodies (1:500; Molecular Probes, Leyden, The Netherlands) were used. Samples were subsequently incubated with DAPI (1:10000, Roche) for 10 min, washed with PBS and mounted in Mowiol. All immunofluorescence data were obtained in a blinded manner by independent observers in a minimum of 6 correlative slices from 1-in-10 series located between −0.4 to +1.6 mm to bregma. Neurodegeneration and glial activation was determined by dopamine- and cAMP-regulated phosphoprotein of 32 kDa (DARPP32; BD Transduction Laboratories, Lexington, KY), Iba-1 (Wako Pure Chemical, Osaka, Japan) and GFAP-Cy3 (Sigma, St. Louis, MO) immunostaining, and quantified with Image-J software. Confocal fluorescence images were acquired by using Leica TCS-SP2 software (Wetzlar, Germany) and SP2 microscope with 2 passes by Kalman filter and a 1024 × 1024 collection box. In the case of 3NP model, 5-μm-thick sections were used for Cresyl-violet staining (Nissl staining) and for immunohistochemical analysis for NeuN, Iba-1 and GFAP as previously described^7^. A Leica DM 2500 microscope and a Leica DFC 420C camera (Leica Microsystems IR GmbH) were used for slide observation and photograph. Staining was quantified using the Image J software designed by the National Institutes of Health (NIH; Bethesda, MD, USA).

### CB_1_ and CB_2_ binding assays

CB_1_ and CB_2_ receptor binding affinities were determined by competition studies using [^3^H]CP55940 as radioligand and commercial membrane preparations of HEK293 EBNA cells stably expressing the respective receptor type (Perkin-Elmer Life and Analytical Sciences, Boston, MA), following a procedure previously described^47^.

### Statistical analysis

*In vitro* data are expressed as mean ± S.D. and *in vivo* results are represented as mean ± SEM. Data were subjected to Kolmogorov-Smirnov normality test and then, differences were analyzed by one-way ANOVA followed by Tukey post hoc test. When data were not normally distributed, significant differences were studied using the Kruskall-Wallis followed by Dunns post-hoc test. *P* < 0.05 was considered significant. Statistical analysis was performed using GraphPad Prism version 5.01. Images were analyzed and quantified using the ImageJ.

## Additional Information

**How to cite this article**: Díaz-Alonso, J. *et al*. VCE-003.2, a novel cannabigerol derivative, enhances neuronal progenitor cell survival and alleviates symptomatology in murine models of Huntington’s disease. *Sci. Rep.* **6**, 29789; doi: 10.1038/srep29789 (2016).

## Supplementary Material

Supplementary Information

## Figures and Tables

**Table 1:** Oxidative stress parameters and proinflammatory markers in the striatum of 3-nitropropionic acid (3NP)-treated mice.

ROUP	COX-2 (2expΔΔct)	TNF-α (2expΔΔct)	IL-6 (2expΔΔct)	%Catalase activity	% GSH	% SOD activity
VEH	1.25 ± 0.68	1.03 ± 0.226	1.2 ± 0.4	100	100	100
3NP	4.69 ± 0.09^*^	5.19 ± 0.327^**^	4.28 ± 0.08^*^	58.85 ± 2.17^***^	67.16 ± 5.06	56.56 ± 1.05^**^
VCE-003.2	1.31 ± 0.75	1.15 ± 058	1.14 ± 0.65	99.05 ± 6.1	101.81 ± 14.59	115.62 ± 27.34
3NP + VCE-003.2	2.7 ± 0.07^###^	1.56 ± 0.576^##^	1.79 ± 0.2^###^	88.82 ± 6.75^#^	111.7 ± 12.6	221.18 ± 26.91^#^
3NP + VCE-003.2 + T0070907	8.91 ± 1^†^	3.83 ± 1.17	8.18 ± 2.9	89.08 ± 5.7	121.48 ± 15.29	121.46 ± 37.04
T0070907	1.45 ± 0.82	1.54 ± 0.75	1.57 ± 0.51	92.18 ± 4.1	115.82 ± 11.23	97.61 ± 27.44

Data presented are the percentage of the vehicle-treated control group and are expressed as means ± SEM (n = 6 animals per group). Statistics: *p < 0.05, **p < 0.01, ***p < 0.001 versus Vehicle; ^#^p < 0.05, ^##^p < 0.01, ^###^p < 0.001 versus 3NP; ^†^p < 0.05 versus the 3NP + VCE-003.2 group.
