# Supplementary material for: VCE-003.2, a novel cannabigerol derivative, enhances neuronal progenitor cell survival and alleviates symptomatology in murine models of Huntington’s disease
Source: Sci Rep. 2016 Jul 19;6:29789. doi: 10.1038/srep29789 (PMC4949444; doi:10.1038/srep29789)
Supplement: Supplementary Information [file srep29789-s1.pdf]

Supplemental information

Title: VCE-003.2, a novel cannabigerol derivative, enhances neuronal progenitor cell survival and alleviates symptomatology in murine models of Huntington's disease.

Authors: Javier Diaz-Alonso[1-3#], Juan Paraíso Luna[1-3#], Carmen Navarrete[4#], Carmen del Rio[5], Irene Cantarero[5], Belen Palomares[5], José Aguareles[1-3], Javier Fernandez-Ruiz[1,3,6], María Luz Bellido[4], Federica Pollastro[7], Giovanni Appendino[7], Marco A. Calzado[5], Ismael Galve-Roperh[1-3*], Eduardo Muñoz[5*]

[1]Instituto Ramón y Cajal de Investigación Sanitaria (IRYCIS), Ctra. Colmenar Viejo, km. 9100, Madrid, Spain. [2]Departamento de Bioquímica y Biología Molecular I. Universidad Complutense, Madrid, Spain. [3]Centro de Investigación Biomédica en Red sobre Enfermedades Neurodegenerativas (CIBERNED), Madrid, Spain. [4]VivaCell Biotechnology España, Córdoba, Spain. [5]Instituto Maimónides de Investigación Biomédica de Córdoba, Hospital Universitario Reina Sofía, Departamento de Biología Celular, Fisiología e Inmunología. Universidad de Córdoba, Spain. [6]Instituto Universitario de Investigación en Neuroquímica. Departamento de Bioquímica y Biología Molecular III, Universidad Complutense, Madrid, Spain. [7]Dipartimento di Scienze Farmaceutiche, Università del Piemonte Orientale, Novara, Italy.

[#]Equal contributing authors.

[*]Co-corresponding Authors: Ismael Galve-Roperh, PhD. Departamento de Bioquímica y Biología Molecular I. Universidad Complutense-CIBERNED, Madrid, Spain, Phone: +34 913944668; e-mail: igr@quim.ucm.es. Eduardo Muñoz MD, PhD. Instituto Maimónides de Investigación Biomédica de Córdoba, Avda. Menéndez Pidal s/n. 14004. Córdoba, Spain. Phone: +34 957213766; e-mail: e.munoz@uco.es

VCE-003 synthesis. tBuOK (2.00 g, 17.824 mmol) was added to a solution of Cannabigerol (CBG) (2.00 g, 6.319 mmol) in toluene (400 mL), to give a purple-colored solution. The reaction mixture was stirred at r.t., in an air-opened round bottom flask, and conversion was monitored by TLC analysis (eluent: 10% EtOAc/hexanes). After 2 h, the reaction mixture was washed with HCl (5% aqueous solution, 300 mL) and the aqueous layer was extracted with EtOAc (100 mL). Combined organic layers were dried over Na_2SO_4 (anhydrous), filtered and concentrated. The crude residue was flash chromatographed on SiO_2 (2 to 4% EtOAc/hexanes), to give 1.10 g of VCE-003 [orange-colored solid, yield: 53%].

^{1}H NMR ($CDCl_3$, 300 MHz): δ 6.94 (s, -OH, 1H), 6.45 (s, 1H), 5.13 (br t, *J* = 6.8 Hz, 1H), 5.04 (br t, *J* = 6.8 Hz, 1H), 3.14 (s, *J* = 6.8 Hz, 2H), 2.41 (t, *J* = 7.8 Hz, 2H), 2.09-1.92 (m, 4H), 1.73 (br s, 3H), 1.57 (br s, 3H), ca. 1.52 (m, 2H), 1.38-1.17 (m, 4H), 0.89 (t, *J* = 7.8 Hz, 3H).

VCE-003.2 synthesis. Ethylamine (5.2 mL, 70% solution in H_2O, 65.403 mmol) was added to a solution of VCE-003 (510 mg, 1.543 mmol) in EtOH (50 mL). The reaction mixture was stirred at r.t. for 2 h and was poured into H_2O (120 mL), taken up to pH= 2 with HCl (10% aqueous solution) and extracted with CH_2Cl_2 (2x80 mL). The organic layer was dried over Na_2SO_4 (anhydrous), filtered and concentrated. Crude residue was purified by reverse phase chromatography (30 to 100% CH_3CN/H_2O) to give 435 mg of 2-(3,7-dimethyl-octa-2,6-dienyl)-6-ethylamino-3-hydroxy-5-pentyl-[1,4]benzo-quinone [purple-colored solid, yield: 75%].

^{1}H NMR ($CDCl_3$, 300 MHz) δ ppm: 6.39 (bs, 1H), 5.09 (m, 2H), 3.54 (t, *J* = 6.6 Hz, 2H), 3.05 (d, *J* = 6.6 Hz, 2H), 2.49 (m, 2H), 1.99 (m, 4H), 1.72 (s, 3H), 1.64 (s, 3H), 1.57 (s, 3H), 1.44-1.22 (m, 9H), 0.88 (m, 3H).

Cysteine recovery assay. 10 mg of VCE-003 and a molar equivalent amount of VCE-003.2 were independently dissolved in 1 mL DMSO, and each solution was next treated with an excess (4 mol. equivalents) of cysteamine. After stirring at room temperature for 1 h, the solutions were diluted with water (2 mL) and extracted with hexane- ether 9:1. After evaporation, the solution was taken up in $CDCl_3$ and analyzed by ^{1}H-NMR. While compound VCE-003.2 could be recovered unscathed in an essential quantitative way,

VCE-003 was undetectable in the residue, indicating that it had irreversibly reactive with cysteamine to form polar and not extractable adducts.

Determination of ROS: N2a cells were seeded at $25x10^3$ cells/ml in 96-well plates and incubated with 0.25 μM 5-(and-6)-chloromethyl-2′,7′-dichlorodihydrofluorescein diacetate acetyl ester (CM-H_2DCFDA) for 20 minutes at 37°C. Then, the cells were treated with either VCE-003 or VCE-003.2 for 2 hours. Cells exposed to pro-oxidant tert-Butyl hydroperoxide (TBHP) were used as positive control. H_2DCFDA oxidation was detected using an IncucyteTM Live-Cell Imaging System (Hertfordshire, UK).

Nrf2 transcriptional assay: To study Nrf2 transcriptional activity N2a cells were seeded (10^5/ml) in 24-well plates and transiently transfected with the plasmid NQO1-ARE-Luc using Roti©-Fect following the manufacturer's instructions (Carl Roth, Karlsruhe, Germany). After 24 hours, cells were incubated with the compounds at the concentrations indicated during 6 hours and the luciferase activity was determined using an Autolumat LB 953 (EG&G Berthold, Bad Wildbad, Germany). Protein content was determined by the Bradford method to calculate RLU/μg of protein and the results were expressed as a fold induction over untreated cells.

Table S1. Human primer sequences used in quantitative Polymerase Chain Reactions

Genes	Forward	Reverse
PPAR-γ	5´-gcgattccttcactgatacactg-3´	5´-gagtgggagtggtcttccattac-3´
LPL	5´-ggcgctaccttgagatagagttctg-3´	5´-tgttttctacagggtgctttagatgac-3´
CEBPA	5´-ccttgtgccttggaaatgcaaac-3´	5´-ctgctcccctccttctctca-3´
ADIPOQ	5´-catgaccaggaaaccacgactc-3´	5´-ccgatgtctcccttaggacca-3
FABP-4	5´-ccaggaatttgacgaagt-3´	5´-tctctttatggtggttgatt-3´
Runx2	5´-tggttaatctccgcaggtcac-3´	5´-actgtgctgaagaggctgtttg-3´
SP7	5´-agccagaagctgtgaaacctc-3´	5´-agctgcaagctctccataacc-3´
ALP	5´-ccaacgtggctaagaatgtcatc-3´	5´-tgggcattggtgttgtacgtc-3´
IBSP	5´-agggcagtagtgactcatccg-3´	5´-cgtcctctccatagcccagtgttg-3´
HPRT	5´-atgggaggccatcacattgt-3´	5´-atgtaatccagcaggtcagca-3´

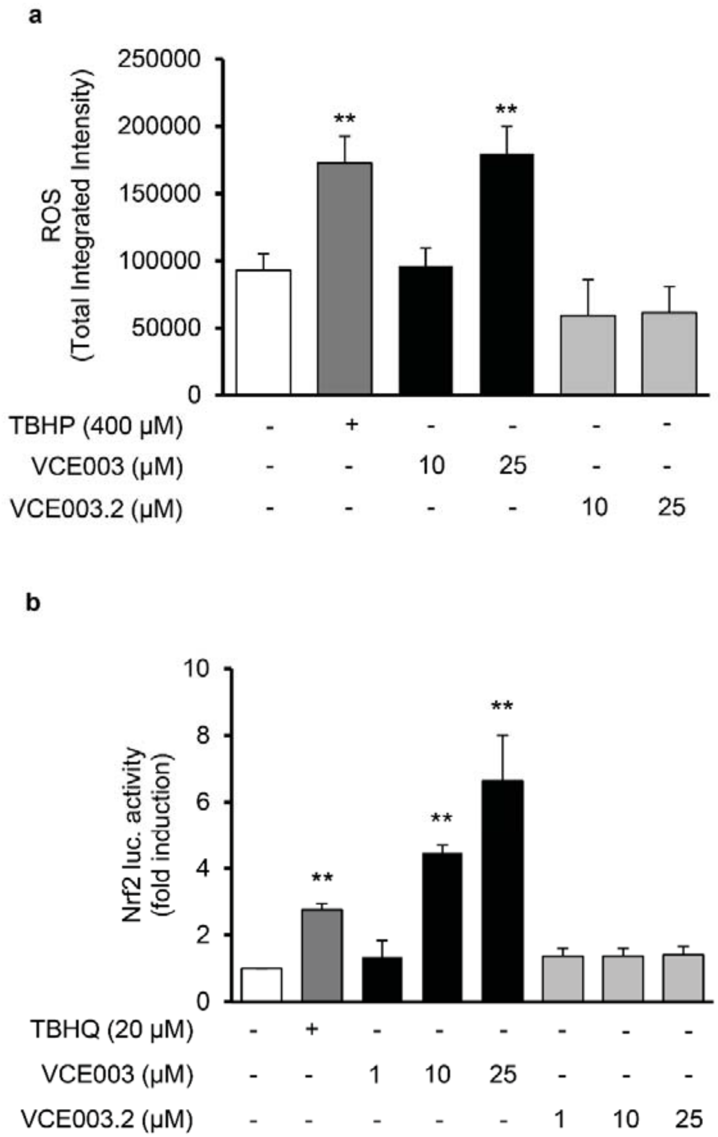

Supplementary Figure S1. Non-electrophilic activity for VCE-003.2. (a) Reactive oxygen species production. N2a cells were stained with CM-H2DCFDA and treated with VCE-003 or VCE-003.2 for 2 hours. Then, reactive oxygen species were measured by Incucyte^{TM} Live-Cell Imaging System. Results are expressed as mean of total integrated intensity (ROS positive cells) ± S.D. **(b)** Effect on Nrf2 transcriptional activity. N2a cells were transiently transfected with the NQO1-ARE-Luc plasmid and incubated with the indicated compound concentrations for 6 hours prior to luciferase activity determination. Results are expressed as the fold induction ± S.D. relative to untreated control. All the results are representative of at least three independent experiments. Statistics: **p<0.01 VCE-003 treatment versus Vehicle treated cells.

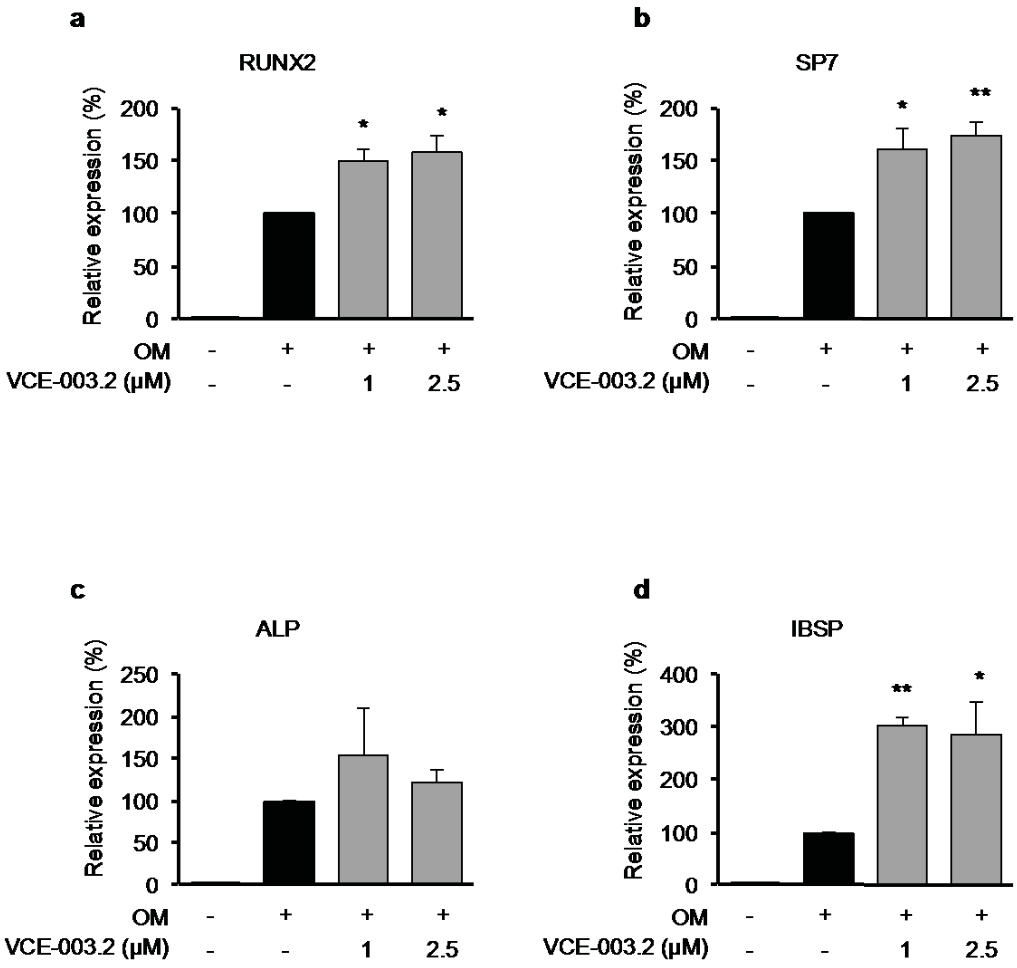

Supplementary Figure S2. Effect of VCE-003.2 on osteoblast-differentiating MSCs. The influence of VCE-003.2 in osteoblastogenesis was evaluated by the changes in the expression of the indicated genes involved in osteoblast formation at 14 days of differentiation. VCE-003.2 caused a mild induction of these genes. Statistics: * $p<0.05$ and ** $p<0.01$ VCE-003.2 treatment vs osteoblast medium (OM).

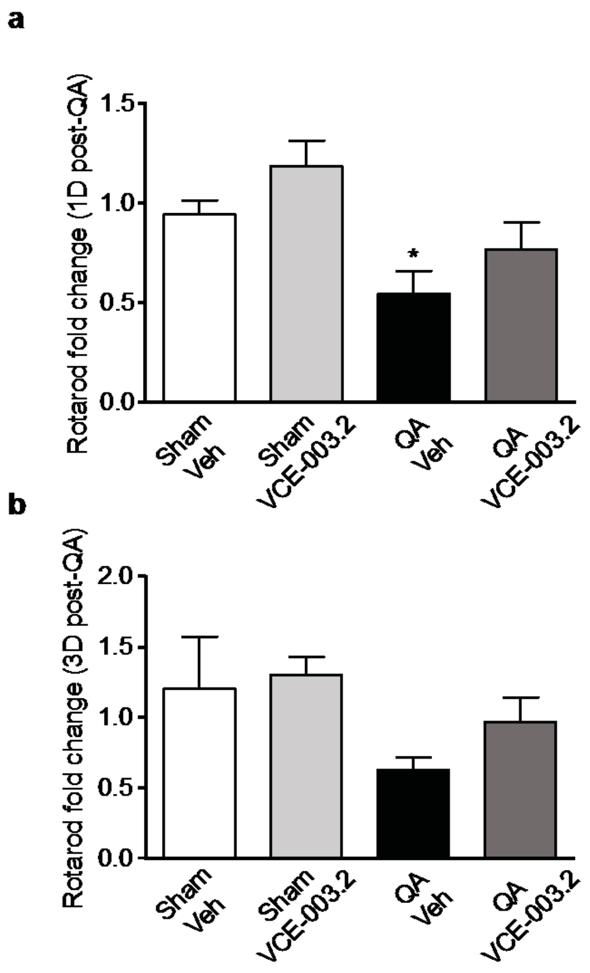

Supplementary Figure S3. Effect of VCE-003.2 administration in QA-induced excitotoxicity *in vivo*. Rotarod performance (fold change) was determined 1 and 3 days after lesion in QA-injected mice treated with vehicle or VCE-003.2. Values are expressed as means ± SEM. Sham-Veh and Sham-VCE (n=7, each group); QA-Veh (n=8); QA-VCE (n=7). Statistics: * p<0.05 versus Sham-Vehicle treated mice.
